# Molecular characterization and genetic diversity studies of Indian soybean (*Glycine max* (L.) Merr.) cultivars using SSR markers

**DOI:** 10.1007/s11033-021-07030-4

**Published:** 2021-12-11

**Authors:** S. P. Jeevan Kumar, C. Susmita, K. V. Sripathy, Dinesh K. Agarwal, Govind Pal, Arvind Nath Singh, Sanjay Kumar, Abhishek Kumar Rai, Jesus Simal-Gandara

**Affiliations:** 1 ICAR-Indian Institute of Seed Science, Mau, Kushmaur, Uttar Pradesh 275103 India; 2 ICAR-Directorate of Floricultural Research, Pune, Maharashtra 411 036 India; 3 Nutrition and Bromatology Group, Analytical Chemistry and Food Science Department, Faculty of Science, Universidade de Vigo, 32004 Ourense, Spain

**Keywords:** Genetic diversity, Heterosis, Hybridization, Polymorphism, SSR markers, Yield

## Abstract

**Background:**

The genetic base of soybean cultivars in India has been reported to be extremely narrow, due to repeated use of few selected and elite genotypes as parents in the breeding programmes. This ultimately led to the reduction of genetic variability among existing soybean cultivars and stagnation in crop yield. Thus in order to enhance production and productivity of soybean, broadening of genetic base and exploring untapped valuable genetic diversity has become quite indispensable. This could be successfully accomplished through molecular characterization of soybean genotypes using various DNA based markers. Hence, an attempt was made to study the molecular divergence and relatedness among 29 genotypes of soybean using SSR markers.

**Methods and results:**

A total of 35 SSR primers were deployed to study the genetic divergence among 29 genotypes of soybean. Among them, 14 primer pairs were found to be polymorphic producing a total of 34 polymorphic alleles; and the allele number for each locus ranged from two to four with an average of 2.43 alleles per primer pair. Polymorphic information content (PIC) values of SSRs ranged from 0.064 to 0.689 with an average of 0.331. The dendrogram constructed based on dissimilarity indices clustered the 29 genotypes into two major groups and four sub-groups. Similarly, principal coordinate analysis grouped the genotypes into four major groups that exactly corresponded to the clustering of genotypes among four sub-groups of dendrogram. Besides, the study has reported eight unique and two rare alleles that could be potentially utilized for genetic purity analysis and cultivar identification in soybean.

**Conclusion:**

In the present investigation, two major clusters were reported and grouping of large number of genotypes in each cluster indicated high degree of genetic resemblance and narrow genetic base among the genotypes used in the study. With respect to the primers used in the study, the values of PIC and other related parameters revealed that the selected SSR markers are moderately informative and could be potentially utilized for diversity analysis of soybean. The clustering pattern of dendrogram constructed based on SSR loci profile displayed good agreement with the cultivar’s pedigree information. High level of genetic similarity observed among the genotypes from the present study necessitates the inclusion of wild relatives, land races and traditional cultivars in future soybean breeding programmes to widen the crop gene pool. Thus, hybridization among diverse gene pool could result in more heterotic combinations ultimately enhancing genetic gain, crop yield and resistance to various stress factors.

**Supplementary Information:**

The online version contains supplementary material available at 10.1007/s11033-021-07030-4.

## Introduction

Soybean (*Glycine max* (L.) Merr.) is one of the world’s most important economic legume crops and second most important oilseed crop of India. It serves as a rich source of oil and protein (20% and 40%) for both human food and animal feed [1, 2]. Among the various soybean growing countries in the world, USA stands first in both production and productivity with 96.62 m.t. and 3157 kg/ha respectively from 306.03 lakh ha of area. Whereas, India in the fifth position produces 9.00 m.t. with productivity of 800 kg/ha from 112.5 lakh ha of area [3]. Although during past few decades, the trend observed with respect to production and productivity revealed remarkable and satisfactory increase in other major soybean growing countries, India is still lagging far behind in productivity due to stagnated yields [4]. In India, even though there is momentous increase in the area and production of soybean during last three decades through the adoption of new varieties, the crop yield potential remained static and becoming major concern among researchers [4, 5]. This could be attributed to the narrow genetic base of soybean cultivars that is either inherited from the crop genetic architecture (self-pollination) or due to the extensive use of selected genotypes as parental lines in the breeding programmes [6]. The targeted hybridization in consequence, led to genetic uniformity and further shrinks in the genetic base of the soybean germplasm, compromising the yield besides enhancing the susceptibility to several biotic and abiotic stresses [7]. Therefore, understanding the genetic diversity of Indian soybean germplasm is critical to explore the untapped valuable genetic traits for enhancing soybean production and productivity. Even though, genetic similarity may not necessarily turn into immediate epidemic, more divergent genetic background is always a key requisite to ensure defense against the unanticipated outbreak of pests and diseases [4, 8].

Diversity in plant genetic resources often enhances the opportunity of plant breeders to breed new and improved cultivars with desirable characteristics [9]. Thus, information on genetic diversity of soybean genotypes could obviously help breeders and geneticists to interpret the germplasm architecture, facilitate the selection of parents with higher levels of diversity, predict superior combinations that deliver best off-spring and accelerate in broadening the genetic base [10]. The assessment of genetic diversity within and between populations is routinely performed based on morphological characterization, biochemical markers and using various molecular marker techniques [11]. Among these, morphological and biochemical markers were identified to be profoundly influenced by environment and several other factors, hence the results are less reproducible with unreliable or biased estimates [12]. Deployment of DNA based marker systems serve as an alternative strategy for precisely discriminating closely related species and cultivars [13, 14]. They work by highlighting differences within the nucleotide sequence between different individuals and remain insensitive to environmental factors [15]. Molecular markers can be broadly classified into two groups based on (i) method of detection as (a) non-PCR derived or hybridization-based techniques (RFLP-Restriction Fragment Length Polymorphism, VNTRs-Variable Number of Tandem Repeats) (b) PCR-derived or amplification-based techniques (RAPD-Random Amplified Polymorphic DNA, AFLP-Amplified Fragment Length Polymorphism, STMSs-Sequence Tagged Microsatellites, SCARs-Sequence Characterized Amplified Regions, CAPS-Cleaved Amplified Polymorphic Sequences, SSLPs-Simple Sequence Length Polymorphisms, Microsatellites or SSRs-Simple Sequence Repeats) and (c) sequence-based markers (SNPs-Single Nucleotide Polymorphisms, DArT- Diverse Array Technology), (ii) mode of gene action as (a) dominant marker (RAPD, AFLP etc.) and (b) co-dominant marker (CAPS, SCAR, SSR etc.) [16].

Among the various listed DNA markers-PCR based, SSRs have demonstrated highest rate of polymorphism and have much greater competence in identifying unique alleles among elite soybean germplasm compared to other marker systems [10–12]. SSRs contain sequences of short tandem repeats distributed over the genomes that are hyper-variable enabling them as an excellent tool for pedigree analysis, genotype differentiation, evaluation of genetic distances or relatedness among genotypes and varietal identification [17–19]. Nevertheless, SNPs have been widely reported as the most abundant class of DNA markers and possess low rates of recurrent mutations that make them evolutionarily stable. They serve as excellent markers for dissecting complex genetic traits and for studying the genomic evolution patterns [20]. In this view, SNPs could serve as an alternative to SSRs for analysis of genetic diversity; however their biallelic nature, low information content and high cost make SSRs still as markers of choice for conducting genetic diversity studies in many crop species [15]. Supporting this, study conducted on comparative genetic diversity analysis using SNPs, DArT and SSRs on sugar beet cultivars revealed that, the success rate was highest for SSR markers owing to their highly polymorphic nature [21, 22]. Precisely, many studies documented deployment of SSR markers to be highly productive for estimation of genetic diversity and relationships among soybean genotypes [12, 17–28]. However, beyond doubt this might be challenged in the coming future with the development of cheap methods for the assay of SNPs.

Annually, several breeding lines and varieties of soybean are developed through selection and hybridization programmes across the globe. Presently, there are more than 100 extant varieties of soybean cultivated in India. Nevertheless, the success of these high yielding and improved varieties largely relies on the availability of quality seed with greater genetic purity standards [29, 30]. The genetic purity of commercial seed lots is traditionally assayed by performing Grow Out Tests (GOT) based on morphological characters that is not only time taking and quite laborious, but also highly environmental responsive [24]. Hence, SSRs are widely deployed for rapid genetic purity assessment and identification of both varieties and hybrids in soybean [25–27]. Keeping this in view, the present investigation was carried out with two objectives i) to study the genetic diversity among 29 genotypes of soybean using selected hyper-variable polymorphic SSR markers and ii) to explore unique and rare alleles that would be useful for genetic purity analysis and varietal identification of soybean.

## Materials and methods

### Plant material

A total of 29 improved and cultivated genotypes/varieties of soybean were obtained from different breeding centers across India and used in the present study. The varieties selected in this study represent a large range of varieties grown in India and most of them  are notified and released for cultivation across different agro-climatic zones of India. Detailed information on pedigree and distinguishable characteristics of all the 29 genotypes are presented in Table 1.

**Table 1:** List of soybean genotypes used in the study and their distinguishing characteristics

Breeding center	Variety	Pedigree/characteristics
Jawaharlal Nehru Krishi Vishwavidyalaya (JNKVV), Jabalpur	Kalitur	Indian landrace
	JS 335	JS 78-77 × JS 71-05 Early duration, high yielding variety, resistant to lodging and shattering
	JS 97-52	PK 327 × L 129 High yielding, multiple disease resistant variety
	JS 76-205	Kalitur × Bragg Semi-determinate, disease resistant variety
	JS 95-60	Selection from PS 73-22 Extra-early cultivar with four seeded pods
	JS 20-69	JS 97-52 × SL 710 High yielding, multiple disease resistant variety
	JS 80-21	JS 75-1 × PK 73-94 Medium duration variety with good longevity
	JS 20-34	JS 98-63 × PK 768 Extra-early, thermo-insensitive, multiple disease resistant
	JS 93-05	Secondary selection from PS 73-22 Resistant to major pests and diseases
	JS 20-98	JS 97-52 × SL 710 High yielding, multiple disease resistant
Indian Institute of Soybean Research (IISR), Indore	NRC 105	Vegetable poded variety, high yielding
	NRC 130	High yielding, moderately resistant to blight
	NRC 131	Moderately resistant to pod blight complex
	NRC 37	Gaurav x Punjab 1 Semi-determinate, high yielding
	NRC 86	RKS 15 × EC 481309 Moderately resistant to major pests and diseases
	NRC 7	Selection from S 69-96 Drought resistant, seeds contain high oil content
Rajmata Vijayaraje Scindia Krishi Vishwavidyalaya (RVSKVV), Gwalior	RVS 2001-4	JS 93-01 × EC 390981 Semi-determinate type, tolerant to major diseases
	RVS 2001-18	Moderately resistant to major pests
Maharashtra Association for the Cultivation of Science (MACS), Pune	MACS 450	Bragg × MACS 111 Semi-determinate type, resistant to YMV and bacterial diseases
	Type 49	Indian landrace
Marathwada Agricultural University (MAU), Parbhani	MAUS 71	JS 71-05 × JS 87-38 Semi-determinate type, yellow color seeds
	MAUS 61	JS 71-1 × PK73-94 Semi-determinate type, resistant to Myrothecium leaf spot
Govind Ballabh Pant University of Agriculture and Technology (GBPUAT), Pantnagar	Shilajeet	Variant discovered from EC 9309
	PS 1092	PK 327 × PK 146 Determinate type, resistant to major foliar diseases
	PK 472	Hardee × Punjab 1 Determinate and high yielding variety
Punjab Agricultural University (PAU), Ludhiana	SL 525	PK 416 × PK 1023 Determinate type, resistant to major diseases especially YMV
University of Agricultural Sciences (UAS), Bengaluru	Karune	Vegetable poded variety
Indira Gandhi Krishi Viswavidhyalaya (IGKV), Raipur	Indira Soya 9	Secondary selection from JS 80-21 Semi-determinate, rust resistant variety
Zonal Agricultural Research Station (ZARS), Kota	RKS 24	PK 472 × PK 1024 Determinate type, moderately resistant to major diseases and pests

### DNA isolation and PCR amplification

Genomic DNA was extracted from seeds using DNeasy® Plant Mini Kit (Qiagen, USA) as per the manufacturer’s instructions. The concentration and quality of the DNA samples was estimated using NanoDrop 2000™ spectrophotometer (Thermo Fisher Scientific, USA). All other chemicals used for DNA extraction and amplification were purchased from Sigma-Aldrich, Germany. Finally, all the genomic DNA samples were diluted to a final concentration of 20 ng µL^−1^ with 1X TE buffer (10 mM Tris-HC1, pH 8.0; 1 mM EDTA) and stored at − 20 °C for further use. Polymerase chain reaction (PCR) amplification was conducted using 25 µL volume mixture containing 1X PCR assay buffer (50 mM KCl, 10 mM Tris-Cl, 1.5 mM MgCl_2_), 200 µM each of dNTPs, 0.2 µM each of forward and reverse primers, 0.6 U *Taq* DNA polymerase and 25 ng of genomic DNA. All PCR reactions were carried out in a thermal cycler AG 22331. Thermal profiling was set up with an initial denaturation at 94 °C for 5 min followed by 33 cycles of denaturation (94 °C for 1 min), annealing (55 °C for 1 min), primer extension (72 °C for 2 min) and a final extension step (72 °C for 7 min).

Amplified PCR products were separated by electrophoresis on 3% (w/v) Metaphor™ agarose gel, stained with ethidium bromide (1 mg/mL) and photographed under UV light using Image Lab™ software. The size of the amplified products was determined using 50 bp DNA ladder as size standard. SSR markers developed by Cregan et al. [32] were used in the present study. A total of 35 SSR markers representing all the 20 linkage groups of soybean were chosen for genotyping from SSR database (http://www.soybase.org) and presented in supplementary Table 1.

### SSR allele scoring and data analysis

The presence or absence of SSR fragment in each genotype was recorded for all the polymorphic SSR primers. Bands appearing without ambiguity were scored as 1 (present) and 0 (absent) for each primer pair. The size of the amplicon was calculated on the basis of band mobility relative to the molecular mass of the ladder. The polymorphic information content (PIC) and expected heterozygosity (H) values reflect the discriminating ability of the marker depending on the number of known alleles and their frequency distribution, thus being alike to genetic diversity; and calculated using the formula given by Botstein et al. [33] in Eq. 1 and Liu [34] in Eq. 2 respectively.

1 $$PIC = {1 }{-} \, \Sigma p_{{\text{i}}}^{{2}} {-} \, \Sigma \, \Sigma p_{{\text{i}}}^{{2}} p_{{\text{j}}}$$

*p*_i_ and *p*_j_ denote the population frequency of the ith and jth alleles. The first summation is over the total number of alleles, whereas the two subsequent summations denote all the i and j where i* ≠ *j*.*

2 $$H = {1 }{-} \, \Sigma p_{{\text{i}}}^{{2}}$$

where, *p*_i_ is the frequency of ith allele in the set of genotypes analysed and calculated for each SSR locus. Effective multiplex ratio (EMR) was calculated as total number of polymorphic loci per primer multiplied by the rate of polymorphic loci from their total number [35, 36]. Marker index (MI) is a statistical parameter used to estimate total utility of the maker system. MI, a product of PIC and EMR was calculated as per Powell et al. [35]. Resolving power (R_p_) is a parameter used to characterize the ability of the primer combination to detect the differences between a large number of genotypes and was calculated according to Prevost and Wilkinson [37].

Phylogenetic tree was constructed from genotyping data of selected polymorphic SSR markers using DARwin software (version 6.0.21) [38] on the basis of genetic distances. The genetic similarity among genotypes was estimated from the dissimilarity (distance) matrix generated from simple matching coefficient. The resulting dissimilarity matrix was further analysed using the unweighted pair-group method arithmetic average (UPGMA) clustering algorithm for construction of a dendrogram. Similarly, neighbor-joining tree was also constructed based on the dissimilarity matrix using unweighed-neighbor joining algorithm from DARwin software (version 6.0.21) [38]. The robustness of the node of the neighbour-joining tree was assessed from 1000 bootstrap replicates and bootstrap values of > 50% were displayed. Principal Coordinates Analysis (PCoA) is a multidimensional scaling (MDS) method used to explore and visualize similarities or dissimilarities in the dataset. It uses either similarity matrix or dissimilarity matrix obtained from original variables and assigns each variable a specific location in a low-dimensional space. In the present study, PCoA was performed to identify similarity indices between the varieties based on Eucledian distance using Past software (version 4.02) [39].

## Results

### SSR polymorphism

A total of 29 promising varieties of soybean were analysed in the present study using 35 crop specific microsatellite markers. Among the 35 SSR primer pairs, 14 primer pairs produced scorable and clear-cut bands and were found to be polymorphic. A total of 48 alleles were detected from these 14 primer pairs of which, 34 alleles were polymorphic with an average of 2.43 alleles per primer pair. The number of alleles generated from each primer pair ranged from 2 (nine primer pairs) to 4 (Satt440). The overall size of the PCR products amplified from these 14 primer pairs ranged from 10 to 180 bp. With respect to allelic frequency, among the 34 polymorphic alleles detected the frequency of 10 alleles (29.41%) was less than 0.25, whereas other 12 alleles (35.29%) had allelic frequency of more than 0.25 but not exceeding 0.5. While seven alleles (20.58%) had frequency of more than 0.50 but less than 0.75, rest of the five alleles (14.71%) had frequency ranging between 0.75 to 1.0. The highest allelic frequency of 0.965 was observed for a single allele using marker Satt-288 and lowest frequency of 0.034 was detected for two alleles using Satt406 and Satt288. The highest PIC value (0.689) was observed in Satt440 and lowest value (0.064) was recorded for the primer Satt288 with average of 0.331. The H values for the markers ranged from 0.067 (Satt288) to 0.738 (Satt440) with an average of 0.401. The EMR values ranged from 0.80 (Satt_243) to 4.0 (Satt440) with an average of 1.89. The MI values for the polymorphic markers varied from 0.055 (Satt_243) to 0.955 (Satt264) with an average of 0.706. R_p_ values for the selected polymorphic primer pairs ranged from 1.793 (Satt431) to 2.206 (Satt440) with an average of 1.966. The respective values for overall genetic variability based on allelic diversity, PIC, H, R_p,_ EMR and MI for all the genotypes are presented in Table 2.

**Table 2:** Allelic status and polymorphism statistics for various polymorphic markers used in the study

Primer	Number of amplified bands	Number of polymorphic bands	Allele size		Highest frequency allele size (bp)	Allelic frequency	PIC	H	EMR	MI	R_P_
			Range	Difference							
Satt406	3	3	100–150	50	150	0.034, 0.172, 0.793	0.301	0.340	3.00	0.9030	2.0000
Satt440	4	4	70–120	50	80, 100, 120	0.156, 0.281, 0.281, 0.281	0.689	0.738	4.00	2.7560	2.2069
Satt562	3	2	10–30	20	10	0.517, 0.482	0.375	0.499	1.33	0.5000	2.0000
Satt_243	5	2	120–140	20	120	0.962, 0.367	0.069	0.071	0.80	0.0552	1.8621
Satt244	4	2	70–90	20	90	0.482, 0.517	0.375	0.499	1.00	0.3750	2.0000
Satt245	5	3	70–100	30	100	0.074, 0.481, 0.444	0.469	0.565	1.80	0.8442	1.8621
Satt264	4	3	100–180	80	180	0.51, 0.444, 0.037	0.425	0.532	2.25	0.9563	1.8621
Satt269	2	2	100–180	80	170	0.724, 0.275	0.320	0.400	2.00	0.6400	2.0000
Satt285	4	2	90–100	10	100	0.071, 0.928	0.124	0.133	1.00	0.1240	1.9310
Satt288	2	2	80–100	20	80	0.965, 0.034	0.064	0.067	2.00	0.1280	2.0000
Satt308	2	2	50–75	25	50	0.517, 0.482	0.375	0.499	2.00	0.7500	2.0000
Satt337	2	2	10–50	40	50	0.793, 0.206	0.274	0.328	2.00	0.5480	2.0000
Satt_366	4	2	80–90	10	90	0.379, 0.620	0.360	0.471	1.00	0.3600	2.0000
Satt431	4	3	90–120	30	120	0.115, 0.692, 0.192	0.421	0.470	2.25	0.9473	1.7931
Total	48	34	–	–	–	–	-	-	-	-	-
Average	3.43	2.43	10–180	–	–	–	0.331	0.401	1.89	0.706	1.966

*PIC* Polymorphic Information Content, *H* Expected Heterozygosity, *EMR* Effective Multiplex Ratio, *MI* Marker Index, *RP* Resolving Power

### Genetic diversity and relatedness among genotypes

**Fig. 1:**
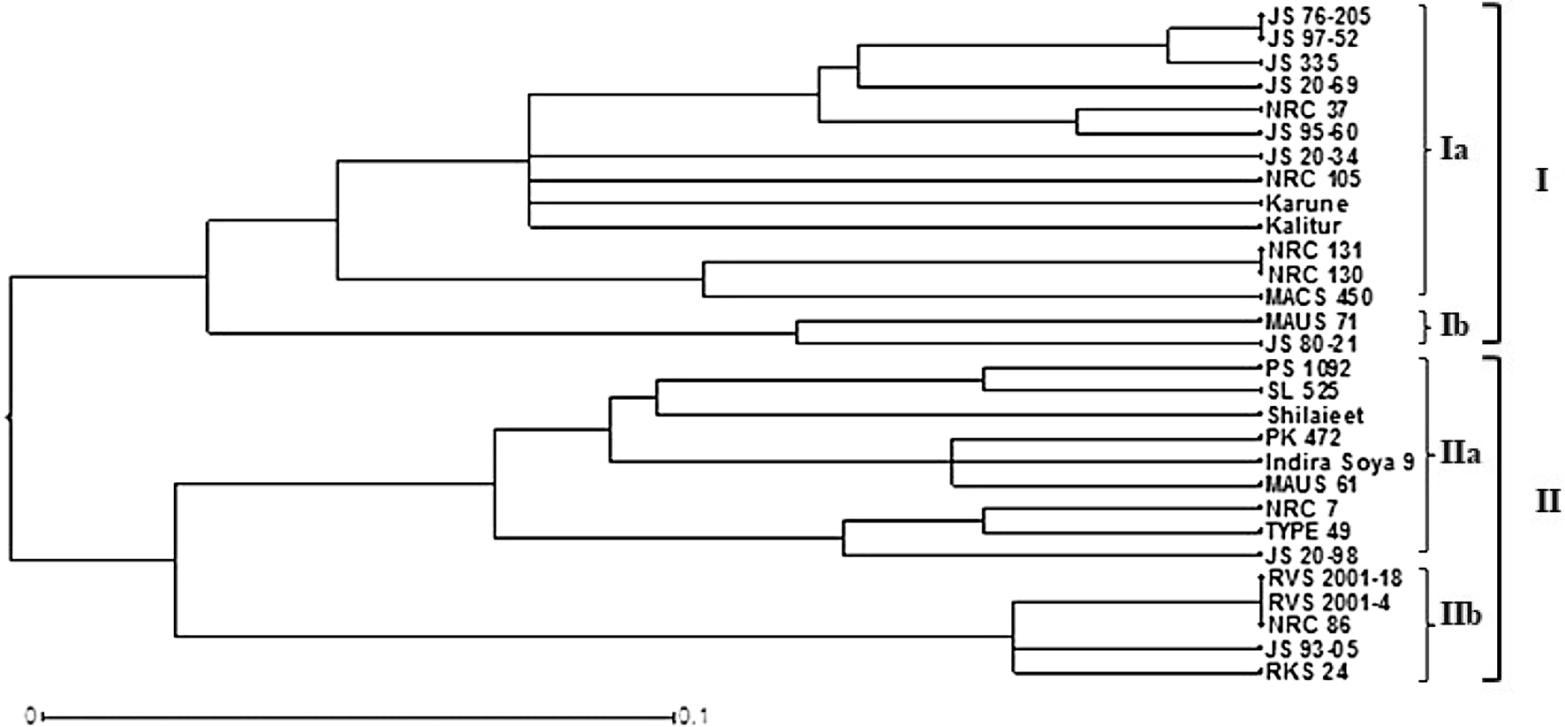
UPGMA dendrogram representing clustering pattern of 29 soybean genotypes

Cluster analysis was performed to elucidate the relationship among the genotypes and the dendrogram is presented in Fig. 1. The pair-wise genetic dissimilarity indices revealed minimum of 0.029 and maximum of 0.676 dissimilarity index between the genotypes. The UPGMA based dendrogram grouped the 29 genotypes of soybean into two major clusters viz*.,* I and II comprising of 15 and 14 genotypes respectively, indicating most of the varieties have parents in common. Cluster I is further divided into two sub-groups Ia and Ib with 13 and 2 genotypes, respectively. Among the 13 genotypes present in sub-group Ia, seven genotypes (JS 76-205, JS 97-52, JS 335, JS 20-69, JS 95-60, JS 20-34, Kalitur) of JNKVV, Jabalpur, four genotypes (NRC 37, NRC 105, NRC 131, NRC 130) of IISR, Indore and one genotype each from UAS, Bengaluru (Karune) and MACS, Pune (MACS 450) were included. The genotypes clustered under this sub-group comprised of land race, selection and hybridization-based varieties. In addition to this, both the vegetable poded soybean genotypes i.e., Karune, NRC 105 developed from Bengaluru and IISR, Indore were included within this sub-group. Sub-group Ib comprised of only two genotypes developed through hybridization and released from two different locations JNKVV, Jabalpur (JS 80-21) and MAU, Parbhani (MAUS 71).

The cluster II with 14 genotypes is also divided into another two sub-groups IIa and IIb comprising of 9 and 5 genotypes, respectively. Among the 9 genotypes in sub-group IIa, all the three genotypes (Shilajeet, PS 1092, PK 472) of GBPUAT, Pantnagar, one genotype each from PAU, Ludhiana (SL 525); IISR, Indore (NRC 7); IGKV, Raipur (Indira Soya 9); MAU, Parbhani (MAUS 61); MACS, Pune (Type 49) and JNKVV, Jabalpur (JS 20-98) were included that comprised of mutant, local land race, selection and hybridization-based varieties. The sub-group IIb with 5 genotypes comprised of one genotype (JS 93-05) developed through secondary selection from JNKVV, Jabalpur, whereas the other 4 genotypes were developed through hybridization and they belong to three different breeding centers viz*.,* IISR, Indore (NRC 86); RVSKVV, Gwalior (RVS 2001-18, RVS 2001-4) and ZARS, Kota (RKS 24).

**Fig. 2:**
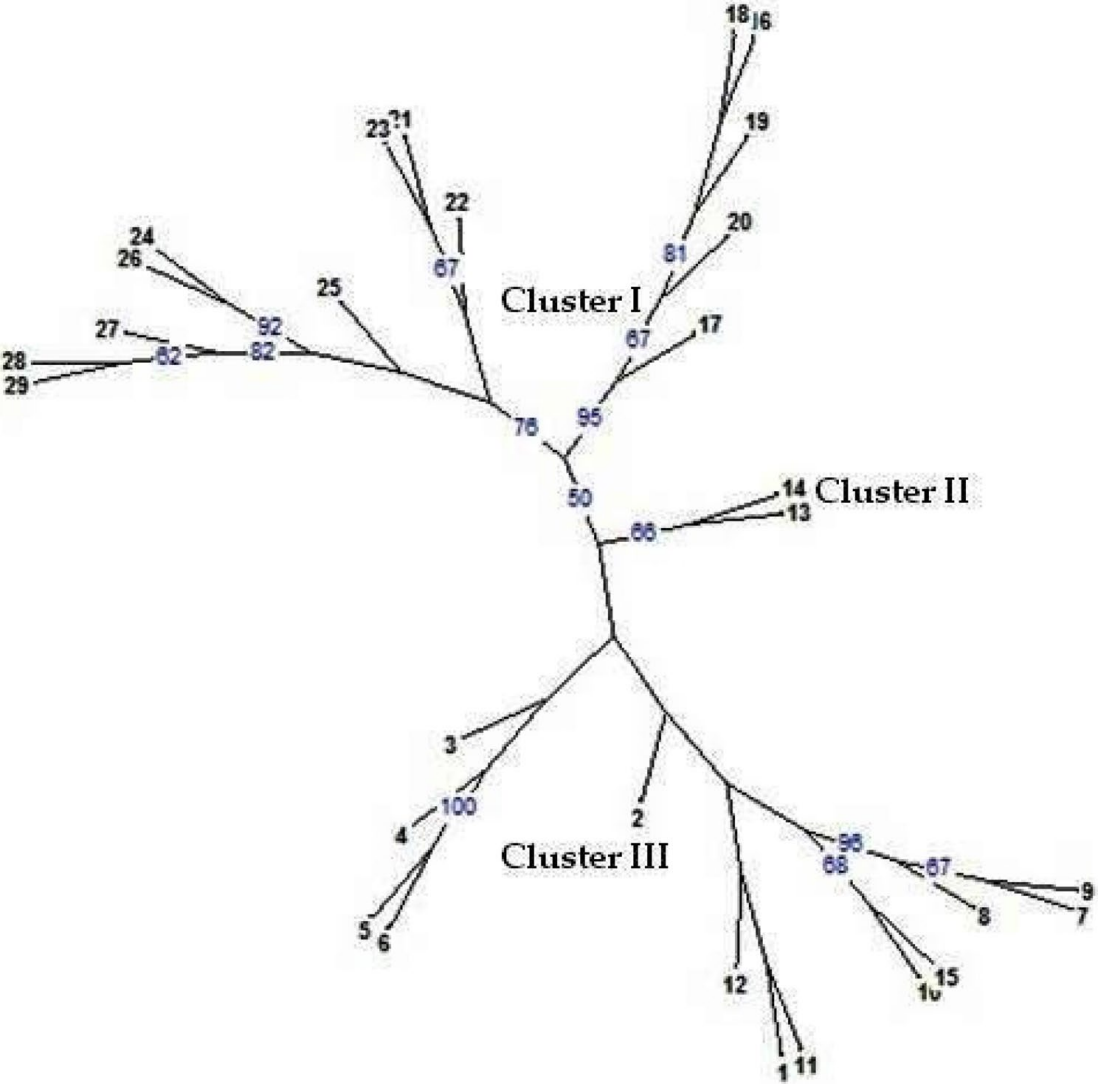
Neighbor-joining tree representing grouping pattern of 29 soybean genotypes [bootstrap values (blue color) of more than 50% are displayed]. The numbers 1 to 29 correspond to the soybean genotypes as follows: 1. Kalitur, 2. Karune, 3. NRC 105, 4. MACS 450, 5. NRC 130, 6. NRC 131, 7. JS 97-52, 8. JS 335, 9. JS 76-205, 10. JS 95-60, 11. JS 20-69, 12. JS 20-34, 13. JS 80-21, 14. MAUS 71, 15. NRC 37, 16. NRC 86, 17. RKS 24, 18. RVS 2001-4, 19. RVS 2001-18, 20. JS 93-05, 21. Type 49, 22. JS 20-98, 23. NRC 7, 24. SL 525, 25. Shilajeet, 26. PS 1092, 27. MAUS 61, 28. Indira Soya 9, 29. PK 472

A neighbor-joining tree (Fig. 2) displaying the genetic-relationships among soybean genotypes was also constructed based on the alleles detected from 14 SSR markers. The genetic distance-based results seen in the neighbor-joining tree revealed three major clusters, resembling the clusters of UPGMA-based dendrogram. The first cluster of neighbor-joining tree comprised of 14 genotypes (Shilajeet, PS 1092, PK 472, SL 525, NRC 7, Indira Soya 9, MAUS 61, Type 49, JS 20-98, JS 93-05, NRC 86, RVS 2001-18, RVS 2001-4, RKS 24), second cluster comprised of only two genotypes (MAUS 71, JS 80-21) and the third cluster had 13 genotypes (JS 76-205, JS 97-52, JS 335, JS 20-69, JS 95-60, JS 20-34, Kalitur, NRC 37, NRC 105, NRC 131, NRC 130, Karune, MACS 450).

**Fig. 3:**
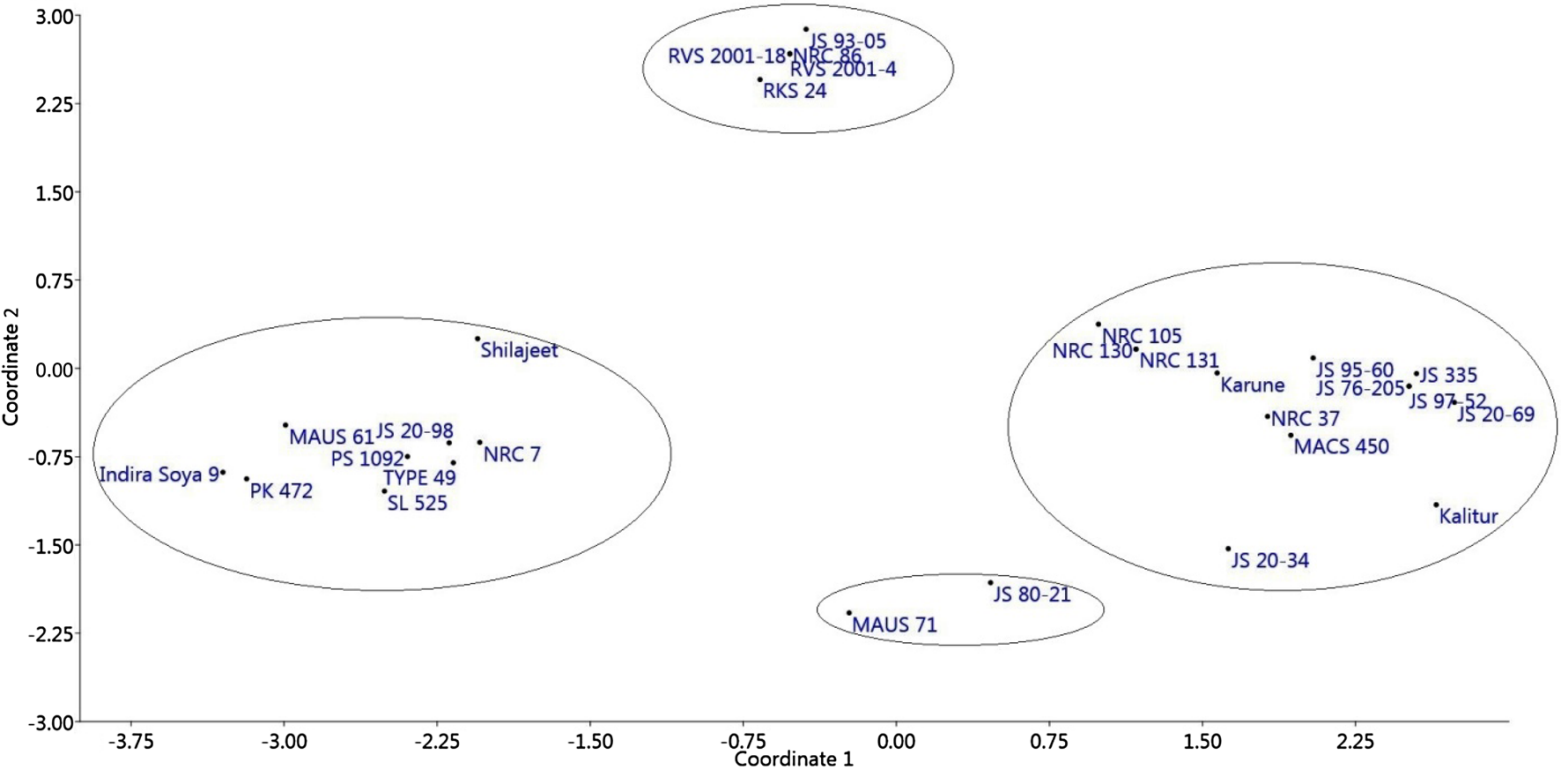
The 2D PCoA clustering of 29 soybean genotypes on the basis of SSR marker data

PCoA was also performed to analyze multi-dimensional relationships that describe the proportion of genetic variance in the dataset used based on the similarity indices (Fig. 3). The scatter plot generated from PCoA clustered the 29 genotypes of soybean into four groups based on similarity indices. The first two principal coordinates (coordinate 1 and coordinate 2) accounted for 34.72% and 15.95% of variation, respectively (based on Eigen values) explaining 50.67% of total variation. Further, the grouping pattern of genotypes is concurrent to the phylogeny-based cluster analysis in the present study.

### Unique alleles

**Fig. 4:**
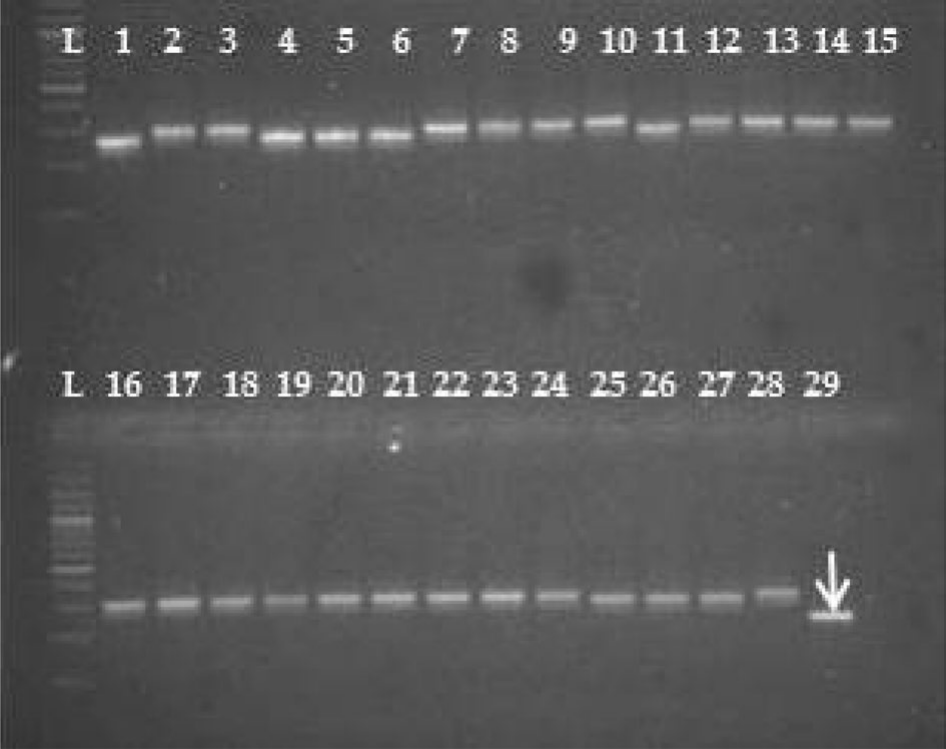
Amplification profile of primer pair Satt406 for 29 soybean cultivars. L-DNA ladder (50 bp). The numbers of lanes 1 to 29 correspond to the soybean cultivars as follows 1. Kalitur, 2. Karune, 3. NRC 105, 4. MACS 450, 5. NRC 130, 6. NRC 131, 7. JS 97-52, 8. JS 335, 9. JS 76-205, 10. JS 95-60, 11. JS 20-69, 12. JS 20-34, 13. JS 80-21, 14. MAUS 71, 15. NRC 37, 16. NRC 86, 17. RKS 24, 18. RVS 2001-4, 19. RVS 2001-18, 20. JS 93-05, 21. Type 49, 22. JS 20-98, 23. NRC 7, 24. SL 525, 25. Shilajeet, 26. PS 1092, 27. MAUS 61, 28. Indira Soya 9, 29. PK 472 (Arrow represents unique allele)

Among the 34 polymorphic alleles identified, eight were detected to be unique alleles generated in specific varieties (Table 3). Satt406 generated unique allele of 100 bp specific to variety PK 472 (Fig. 4). Similarly, Satt288 produced amplicon size of 100 bp in variety SL 525. Satt285 produced two allelic variants of size 90 bp, 100 bp wherein 90 bp was specific for the identification of variety JS 93-05; and both the alleles (90 bp, 100 bp) were amplified in the variety JS 335. Similarly, Satt440 produced two alleles of size 70 bp, 80 bp that could inadvertently distinguish Kalitur from remaining soybean genotypes. Satt264 amplified one allele of size 180 bp that could visually distinguish MAUS 61 from other genotypes. Satt_243 amplified an allele of size 140 bp specific for the variety NRC 7. The eight unique alleles generated by these specific primer pairs are indistinguishable for the identification of seven soybean varieties.

**Table 3:** Unique and rare alleles identified from SSR markers specific to varieties

Primer pair	Size	Variety
Unique alleles		
Satt406	100 bp	PK 472
Satt288	100 bp	SL 525
Satt285	90, 100 bp	JS 335
	90 bp	JS 93-05
Satt264	180 bp	MAUS 61
Satt440	70, 80 bp	Kalitur
Satt_243	140 bp	NRC 7
Rare alleles		
Satt245	70 bp	Kalitur, Karune
Satt431	90 bp	Kalitur, NRC 105, RKS 24

### Rare alleles

As per International Union for the Protection of New Varieties of Plants (UPOV) guidelines, the rare alleles are those present at a specific locus and appear with a frequency below an agreed threshold (commonly 5-10%) and hence they may also be employed in cultivar identification. In the current study, two rare alleles were detected which appeared in two to three varieties. Amlpicon size of 70 bp generated from Satt245 appeared in two varieties Kalitur and Karune, whereas another allele of size 90 bp generated from Satt431 appeared in three varieties viz*.,* Kalitur, NRC 105 and RKS 24.

## Discussion

Deployment of SSR markers for assessment of genetic diversity has been widely adopted for screening of soybean germplasm [40, 41]. In the present study, 14 markers present on six linkage groups of soybean were found to be polymorphic and the high percentage of polymorphic loci (70.8%) detected were consistent with the previous reports [42, 43]. The identified polymorphic SSR primers amplified with an average number of 2.43 alleles per locus and PIC of 0.331. The level of genetic diversity obtained in the current study is in agreement with the findings of few authors, who reported average of 3.23 alleles with PIC value of 0.386 [41], 2.97 alleles with PIC of 0.447 [44], 2.85 alleles with PIC of 0.360 [45], 2.41 alleles with PIC value of 0.437 [31] and 2.22 alleles per SSR locus with PIC of 0.199 [43] in soybean. However, other studies reported high rates of genetic diversity using SSR markers having 4.9 alleles with average PIC of 0.560 [42], 4 alleles with PIC of 0.580 [46], 4 alleles with PIC of 0.590 [47] and 5 alleles with PIC of 0.610 [48]. All these studies reported comparatively higher number of alleles per locus and average PIC values in comparison to the present study.

Most of the SSR markers (10/14) used in this study had PIC values ≥ 0.3 and one marker Satt440 had PIC value of > 0.6 with highest number of alleles (4) that denotes a strong correlation between PIC and allele richness [49]. Therefore, it is anticipated that allelic richness serves as an effective index for diversity evaluation; nevertheless, it largely relies on the sample size [12]. The moderate level of allelic richness and PIC values observed in the present study could also be attributed to the narrow genetic base of the cultivars used for analysis. In the present study, average heterozygosity of 0.401 was reported that is in agreement with the findings of Zhang et al. [41] and Wang et al. [50] who reported average heterozygosity values of 0.460, 0.446 in vegetable and wild types of soybean respectively. Further, the results of MI, EMR and heterozygosity clearly emphasize that the SSR markers selected for the present study are moderately informative and could be utilized for diversity analysis of soybean genotypes.

Both UPGMA dendrogram and neighbor-joining tree are in similarity with each other and the clustering of genotypes is either based on homology in their origin or similarity in the parental material used for breeding programmes [6, 47]. The first cluster of neighbor-joining tree comprised of 14 genotypes resembling cluster II of UPGMA dendrogram. The second cluster comprised of only two genotypes, while the third cluster had 13 genotypes in the neighbor-joining tree. The two genotypes MAUS 71, JS 80-21 that emerged as a separate cluster (cluster II) in neighbor-joining tree were merged within the cluster I of UPGMA dendrogram. However, these two genotypes evolved as a separate sub-group (Ib) under cluster I of UPGMA dendrogram.The pattern of clustering of the remaining genotypes remained same for both UPGMA-dendrogram and neighbor-joining tree. In the present study, all the 29 genotypes of soybean were grouped into two major clusters and various sub-groups in the dendrogram on the basis of their genetic relationships. Similarly, Wang et al. [13] reported two clusters using ten SSR markers, Tantasawat et al. [49] identified four clusters using 11 SSR markers, Ghosh et al. [51] reported two clusters and six sub-clusters from 10 SSR markers, Chauhan et al. [47] obtained two clusters using 21 SSR markers and Hipparagi et al. [45] reported three clusters using 21 SSR markers in soybean.

The genotypes Kalitur, JS 335, JS 97-52, JS 76-205, JS 95-60, JS 20-69, JS 20-34 released from Jabalpur center and NRC 105, NRC 130, NRC 131, NRC 37 developed at Indore center were clustered under the sub-group (Ia) of cluster I of dendrogram on the basis of genetic affinity. Under cluster I, all the varieties developed through hybridization share close affinity with each other due to homology in parental material used for hybridization; and based on the degree of relatedness, the varieties were demarcated into different sub-groups (Ia and Ib) under a single cluster. In agreement to this, two varieties viz*.,* JS 80-21 and MAUS 71 originated from different breeding centers although were grouped under cluster I, they diverged as a separate sub-group (Ib) due to the genetic distances in parental material utilized for hybridization. Interestingly, these two genotypes were demarcated as a separate cluster in both neighbor-joining tree and PCoA grouping.

In case of cluster II, varieties RVS 2001-4, RVS 2001-18, NRC 86, JS 93-05, RKS 24 developed at different breeding centers were clustered under sub-group (IIa) that could be assigned either to homology in parental material used for breeding programme or based on the origin of parent material. Likewise, all the varieties released from Pantnagar viz*.*, Shlajeet, PS 1092, PK 472 and one variety each viz., MAUS 61, NRC 7, Type 49, SL 525 and Indira Soya 9 developed from Parbhani, Indore, Pune, Ludhiana and Raipur centers respectively, clustered under same sub-group (IIb) of cluster II. The findings of this study are supported by the pedigree presented in Table 1. Apart from this, two sets of sister lines viz*.,* JS 95-60, JS 93-05 (developed through selection from PS 73-22) and JS 20-69, JS 20-98 (developed through hybridization from JS 97-52 × SL 710), were grouped into different clusters since they had different genetic profile at these 14 polymorphic loci that clearly reinforce the effectiveness of SSR markers used in the study. The results obtained from the present study clearly demonstrated the potentiality of SSR markers in precise varietal identification, supporting the findings of Chotiyarnwang et al. [52], Tantasawat et al. [49] and Singh et al. [53].

To complement the information obtained from hierarchical cluster analysis, PCoA was performed that again clustered the genotypes into four groups exactly resembling the sub-groups of dendrogram. Comparable to the cluster analysis, PCoA separated the genotypes into four major groups corresponding to the four sub-groups of dendrogram. The PCoA also revealed that most of the soybean genotypes were intermixed into a large group (except MAUS 71, JS 20-81) and exactly corresponding to Ia, Ib, IIa and IIb sub-groups of dendrogram. The results obtained are consistent with the findings of previous reports [12, 13, 42, 47–49, 51, 52, 54, 55]. The results from the present study clearly epitomize that SSR markers could serve as an efficient tool for analysing the genetic diversity among the genotypes and also aid in determining the pedigree relationships in soybean.

The unique or rare alleles generated through natural mutation and selection [56] are often utilized for categorization of germplasm collections, breeding and genetic purity analysis that serve as unique markers [24]. This study reported eight unique alleles amplified from six primer pairs that are specific for the identification of seven varieties and could be potentially utilized for varietal identification and DNA fingerprinting. In congruity to this, Meesang et al. [25] and Zhang et al. [41] have validated the use of SSR markers for genetic purity analysis in different varieties and hybrids of soybean. Analogous to the present study, Tantasawat et al. [49], Sahu et al. [27] and Rani et al. [31] detected unique alleles from their study using SSR markers. Further, the study detected two rare alleles generated from two different markers indistinguishable for identification of 2-3 varieties. Similar results were reported by Rani et al. [31], wherein 11 rare alleles were identified that could potentially identify a set of 2-11 soybean cultivars.

## Conclusion

In the present study, the extent of genetic diversity among the investigated genotypes of soybean was reported to be moderate and distributed over two major clusters as evident from the UPGMA dendrogram. The clustering of large number of genotypes in each single cluster indicated high genetic relatedness among the material used. Further, a good association between genetic divergence among the cultivars based on their origin and pedigree has been noticed. The present study also confirms the hypothesis that narrow genetic base exists among the soybean cultivars of India. In addition to this, the study could identify a set of 14 polymorphic markers that could be inadvertently used for diversity analysis of soybean. Besides, the information on unique and rare alleles obtained from the study could be positively utilized for cultivar identification and genetic purity control in soybean. To explore further the diversity of soybean, utilizing of more SSR markers that cover genome/chromosomes of soybean would be desirable for further studies. In summary, the results from this study make it imperative that widening of soybean genetic base is critically essential to exploit heterosis and overcome yield stagnation. This can be achieved by introduction of new alleles into the future soybean breeding programmes of India by inclusion of more landraces, wild relatives and exotic germplasm lines.

## Supplementary Information

Below is the link to the electronic supplementary material.

Supplementary file1 (DOCX 22 KB)

## References

[1] Ibanda AP, Karungi J, Malinga GM, Adjumati G (2018). Influence of environment on soybean [*Glycine max* (L.) Merr.] resistance to groundnut leaf miner, [(*Aproaerema modicella* (Deventer)] in Uganda. J Plant Breed Crop Sci.

[2] Kumar SPJ, Kumar A, Ramesh KV, Singh C, Agarwal DK, Pal G, Kuchlan MK, Singh R (2020). Wall bound phenolics and total antioxidants in stored seeds of soybean (*Glycine max*) genotypes. Indian J Agric Sci.

[3] USDA Foreign agricultural service (2020) https://www.fas.usda.gov/. Accessed 12 Dec 2020.

[4] Bharadwaj CH, Satyavathi CT, Tiwari SP, Karmakar PG (2002). Genetic base of soybean (*Glycine max*) varieties released in India as revealed by coefficient of parentage. Ind J Agric Sci.

[5] Singh RP, Chintagunta AD, Dinesh KA, Kureel RS, Kumar SPJ (2020). Varietal replacement rate: prospects and challenges for global food security. Glob Food Agric.

[6] Mukuze C, Tukamuhabwa P, Maphosa M, Dari S, Dramadri IO, Obua T, Kongai H, Rubaihayo P (2020). Genetic diversity analysis among soybean genotypes using SSR markers in Uganda. Afr J Biotech.

[7] Sendege G, Obua T, Kawuki R, Maphosa M, Tukamuhabwa TP (2015). Soybean genetic diversity and resistance to soybean rust disease in Uganda. Agric J.

[8] Varshney RK, Kudapa H (2013). Legume biology: the basis for crop improvement. Funct Plant Biol.

[9] Susmita C, Kumar SPJ, Chintagunta AD, Agarwal DK (2021). Apomixis: a foresight from genetic mechanisms to molecular perspectives. Bot Rev.

[10] Kumar A, Ramesh KV, Chandusingh A, Sripathy KV, Dinesh KA, Pal G, Mrinal KK, Singh RK, Ratnaprabha A, Kumar SPJ (2019). Bio-prospecting nutraceuticals from selected soybean skins and cotyledons. Ind J Agric Sci.

[11] Singh C, Kumar SPJ, Sripathy KV, Somasundaram G, Udaya Bhaskar K, Ramesh KV, Kumar M, Prasad SR (2017). Characterization and identification of rice germplasm accessions using chemical tests. Seed Res.

[12] Wang L, Guan R, Zhangxiong L, Chang R, Qiu L (2006). Genetic diversity of Chinese cultivated soybean revealed by SSR markers. Crop Sci.

[13] Wang L, Guan Y, Guan R, Li Y, Ma Y, Dong Z, Liu X, Zhang H, Zhang Y, Liu Z, Chang R, Xu H, Li L, Lin F, Luan W, Yan Z, Ning X, Zhu L, Cui Y, Piao R, Liu Y, Chen P, Qiu L (2006). Establishment of Chinese soybean (*Glycine max*) core collections with agronomic traits and SSR markers. Euphytica.

[14] Kumar SPJ, Susmita C, Agarwal DK, Pal G, Rai AK, Simal-Gandara J (2021). Assessment of genetic purity in rice using polymorphic SSR markers and its economic analysis with Grow-Out-Test. Food Anal Methods.

[15] Mondini L, Noorani A, Pagnotta MA (2009). Assessing plant genetic diversity by molecular tools. Diversity.

[16] Nadeem MA, Nawaz MA, Shahid MQ, Doğan Y, Comertpay G, Yıldız M (2018). DNA molecular markers in plant breeding: current status and recent advancements in genomic selection and genome editing. Biotechnol Biotechnol Equip.

[17] Guan R, Chang R, Li Y, Wang L, Liu Z, Qiu L (2010). Genetic diversity comparison between Chinese and Japanese soybeans (*Glycine max* (L.) Merr.) revealed by nuclear SSRs. Genet Resour Crop Evol.

[18] Wang M, Li R-Z, Yang W-M, Du W-J (2010). Assessing the genetic diversity of cultivars and wild soybeans using SSR markers. Afr J Biotechnol.

[19] Agarwal RK, Brar DS, Nandi S, Huang N, Khush GS (2009). Phylogenetic relationship among Oryza species revealed by AFLP markers. Theor Appl Genet.

[20] Alemu A, Feyissa T, Letta T (2020). Genetic diversity and population structure analysis based on the high density SNP markers in Ethiopian durum wheat (*Triticum turgidum* ssp. durum). BMC Genet.

[21] Simko I, Eujayl I, van Hintum TJL (2012). Empirical evaluation of DArT, SNP and SSR marker-systems for genotyping, clustering, and assigning sugar beet hybrid varieties into populations. Plant Sci.

[22] Zhang CB, Peng WL, Zhang SM, Wang H, Sun Y, Dong S, Zhao LM (2014). Application of SSR markers for purity testing of commercial hybrid soybean (*Glycine max* L.). J Agric Sci Technol.

[23] Liu L, Wang Y (2000). Identification of maize seed purity based on electrophoresis. Seed Word.

[24] Li YC, Korol AB, Fahima T, Beiles A, Nevo E (2002). Microsatellites: genomic distribution, putative functions and mutational mechanisms: a review. Mol Ecol.

[25] Meesang N, Ranamukhaarachchi SL, Petersen MJ, Anderson SB (2001). Soybean cultivar identification and genetic purity analysis using microsatellite DNA markers. Seed Sci Technol.

[26] Tantasawat P, Trongchuen J, Prajongjai T, Jenweerawat S, Chaowiset W (2011). SSR analysis of soybean (*Glycine max* (L.)Merr.) genetic relationship and variety identification in Thailand. AJCS.

[27] Sahu P, Khare D, Tripathi N, Shrivastava AN, Saini N (2012). Molecular screening for disease resistance in soybean. J Food Leg.

[28] Roy S, Dikshit PK, Sherpa KC, Singh A, Jacob S, Rajak RC (2021). Recent nanobiotechnological advancements in lignocellulosic biomass valorization: a review. J Environ Manage.

[29] Agarwal RK, Brar DS, Nandi S, Huang N, Khush N (1999). Phylogenetic relationship among Oryza species revealed by AFLP markers. Theor Appl Genet.

[30] Kumar SPJ, Chintagunta AD, Reddy YM, Rajjou L, Kumar GV, Agarwal DK, Prasad SR, Simal-Gandara J (2021). Implications of reactive oxygen and nitrogen species in seed physiology for sustainable crop productivity under changing climate conditions. Curr Plant Biol.

[31] Rani A, Kumar V, Gill BS, Rathi P, Shukla S, Singh RK, Husain SM (2017). Linkage mapping of Mungbean yellow mosaic India virus (MYMIV) resistance gene in soybean. Breed Sci.

[32] Cregan PB, Jarvik T, Bush AL, Shoemaker RC, Lark KG, Kahler AL, Kaya N, vanToai TT, Lohnes DG, Chung J, Specht JE (1999). An integrated genetic linkage map of the soybean genome. Crop Sci.

[33] Botstein D, White RL, Skalnick MH, Davies RW (1980). Construction of a genetic linkage map in man using restriction fragment length polymorphism. Am J Hum Genet.

[34] Liu BH (1998). Statistical genomics: linkage, mapping and QTL analysis.

[35] Powell W, Morgante M, Andre C, Hanafey M, Vogel J, Tingsey S, Rafalski A (1996). The utility of RFLP, RAPD, AFLP and SSR (microsatellite) markers for germplasm analysis. Mol Breed.

[36] Nagaraju J, Damodar RK, Nagaraja GM, Sethuraman BN (2001). Comparison of multilocus RFLPs and PCR-based marker systems for genetic analysis of the silkworm, *Bombyx mori*. Heredity.

[37] Prevost A, Wilkinson MJ (1999). A new system of comparing PCR primers applied to ISSR fingerprinting of potato cultivars. Theor Appl Genet.

[38] Perrier X, Jacquemoud-Collet JP (2006) DARwin Software. http://darwin.cirad.fr/darwin. Accessed 30 April 2020

[39] Hammer Ø, Harper DAT, Paul DR (2001). Past: paleontological statistics software package for education and data analysis. Palaeontology.

[40] Li Y, Guan R, Liu Z, Ma Y, Wang L, Li L, Lin F, Luan W, Chen P, Yan Z, Guan Y, Zhu L, Ning X, Smulders MJM, Li W, Piao R, Cui Y, Yu Z, Guan M, Chang R, Hou A, Shi A, Zhang B, Zhu S, Qiu L (2008). Genetic structure and diversity of cultivated soybean (*Glycine max* (L.) Merr.) landraces in China. Theor Appl Genet.

[41] Zhang G, Xu S, Mao W, Hu Q, Gong Y (2014). Determination of the genetic diversity of vegetable soybean [*Glycine max* (L.) Merr.] using EST-SSR markers. J Zhejiang Sci B.

[42] Narvel JM, Fehr WR, Chu WC, Grant D, Shoemaker RC (2000). Simple sequence repeat diversity among soybean plant introductions and elite genotypes. Crop Sci.

[43] Bisen A, Khare D, Nair P, Tripathi N (2015). SSR analysis of 38 genotypes of soybean (*Glycine max* (L.)Merr.) genetic diversity in India. Physiol Mol Biol Plant.

[44] Kumawat G, Singh G, Gireesh C, Shivakumar M, Arya M, Agarwal D, Husain S (2015). Molecular characterization and genetic diversity analysis of soybean (*Glycine max* (L.) Merr.) germplasm accessions in India. Physiol Mol Biol Plant.

[45] Hipparagi Y, Singh R, Roy C, Debjani GV (2017). Genetic diversity and population structure analysis of Kala bhat (*Glycine max* (L.) Merrill) genotypes using SSR markers. Hereditas.

[46] Ristova D, Šarčević H, Šimon S, Mihajlov L, Pejić I (2010). Genetic diversity in southeast European soybean germplasm revealed by SSR markers. Agric Conspec Sci.

[47] Chauhan DK, Bhat J, Thakur A, Kumari S, Hussain Z, Satyawathi CT (2015). Molecular characterization and genetic diversity assessment in soybean [*Glycine max* (L.) Merr.] varieties using SSR markers. Int J Curr Microbiol Appl Sci.

[48] Gupta S, Manjaya J (2017). Genetic diversity and population structure of Indian soybean [*Glycine max*(L.) Merr.] revealed by simple sequence repeat markers. J Crop Sci Biotechnol.

[49] Tantasawat P, Trongchuen J, Prajongjai T, Seehalak W, Jittayasothorn Y (2011). Variety identification and comparative analysis of genetic diversity in yard long bean (*Vigna unguiculata* spp. sesquipedalis) using morphological characters, SSR and ISSR analysis. Sci Hort.

[50] Wang YH, Zhang XJ, Fan SJ (2015). Genetic diversity of wild soybean populations in Dongying, China, by simple sequence repeat analysis. Genet Mol Res.

[51] Ghosh J, Ghosh PD, Choudhury PR (2014). An assessment of genetic relatedness between soybean [*Glycine max* (L.) Merrill] cultivars using SSR markers. Am J Plant Sci.

[52] Chotiyarnwong O, Chatwachirawong P, Chanprame S, Srinivas P (2007). Evaluation of genetic diversity in Thai indigenous and recommended soybean varieties by SSR markers. Thai J Agric Sci.

[53] Singh RK, Mishra SK, Singh SP, Mishra N, Sharma ML (2010). Evaluation of microsatellite markers for genetic diversity analysis among sugarcane species and commercial hybrids. Aust J Crop Sci.

[54] Souframanien J, Gopalakrishna T (2004). A comparative analysis of genetic diversity in blackgram genotypes using RAPD and ISSR markers. Theor Appl Genet.

[55] Narvel JM, Chu WC, Fehr WR, Cregan PB, Shoemaker RC (2000). Development of multiplex sets of simple sequence repeat DNA markers covering the soybean genome. Mol Breeding.

[56] Mousadik A, Petit RJ (1996). High level of genetic differentiation for allelic richness among populations of the argan tree [*Argania spinosa*(L.) Skeels] endemic to Morocco. Theor Appl Genet.

