# Supplementary material for: Molecular characterization and genetic diversity studies of Indian soybean (Glycine max (L.) Merr.) cultivars using SSR markers
Source: Mol Biol Rep. 2021 Dec 11;49(3):2129–40. doi: 10.1007/s11033-021-07030-4 (PMC8863763; doi:10.1007/s11033-021-07030-4)
Supplement: Supplementary file 1 — Supplementary file1 (DOCX 22 KB) [file 11033_2021_7030_MOESM1_ESM.docx]

**Supplementary table 1:** SSR markers and their sequences selected for the study

| Primer | Forward sequence | Reverse sequence | Linkage group | Amplification status |
| --- | --- | --- | --- | --- |
| Satt363 | TAG AGG GAT CCA ATT AGA ATC CAA GAA A | TCT TCG ATC TTC ATA TTC ATT TTT GTA A | C2 | M |
| Satt157 | GGG CTC ACT CTC GAT AGT AGG TAT AAA G | GGG ATA CCA AAA GGA ATA ATT GTC TT | D1b | M |
| Sat_243 | GCG TCC AGC CTG ACC ATT TTA | GCG GCA ACC GCT TAA AAA TAA TTT AAG AT | K | P |
| Sat_366 | GCG GCA CAA GAA CAG AGG AAA CTA TT | GCG GAC ATG GTA CAT CTA TAT TAC GAG TAT T | J | P |
| Satt570 | CTC ATG TGG TCC TAC CCA GAC TCA | CGC TAT CCC TTT GTA TTT TCT TTT GC | G | M |
| Satt191 | CGC GAT CAT GTC TCT G | GGG AGT TGG TGT TTT CTT GTG | G | M |
| Satt142 | GGA CAA CAA CAG CGT TTT TAC | TTT GCC ACA AAG TTA ATT AAT GTC | H | M |
| Satt239 | GCG CCA AAA AAT GAA TCA CAA T | GCG AAC ACA ATC AAC ATC CTT GAA C | I | M |
| Satt244 | GCG CCC CAT ATG TTT AAA TTA TAT GGA G | GCG ATG GGG ATA TTT TCT TTA TTA TCA G | J | P |
| Satt245 | AAC GGG AGT AGG ACA TTT TAT T | GCG CCT CCT GAA TTT CAA AGA ATG AAG A | M | P |
| Satt264 | CCT TTT GAC AAT TAT GGC ATA TA | GCA TAG AAG GGC ATC ATT CAG AT | K | P |
| Satt269 | GCG TGC CAG GTA GAA AAA TAT  TAG | GCG GTT TTT CACT TTT CAA AAT TC | K | P |
| Satt285 | GCG ACA TAT TGC ATT AAA AAC ATA CTT | GCG GAC TAA TTC TAT TTT ACA CCA ACA AC | J | P |
| Satt274 | GCG GGG TCA ATT AGT TTT CGT CAG TT | GCG CAC GGT ATA TAA TCG AAC CTA T | D1b | M |
| Satt301 | GCG AAA CAC TCC TAG TTG ATT ACA AA | GCG ATA TAA TGC ACA AAG AAA TTA AAG A | D2 | M |
| Satt077 | GAT CTA AAG TCT GAT ATT TTT AAC TA | AAA AGG AGA AGG AAT GC | D1a | M |
| Satt459 | TCG TGT TAG ATT TTT ACT GTC ACA TT | AAC TGC ATA CCC TTT GTT TGA A | D1b | M |
| Satt230 | CCG TCA CCG TTA ATA AAA TAG CAT | CTC CCC CAA ATT TAA CCT TAA AGA | E | M |
| Satt666 | TGG CTT GTC ATC TCT ACT TTT ATT AG | TCA TGC ATC TAA TTT GTT TTA TCT ATC A | H | NA |
| Satt384 | TGG GGG TCA ATT TTA ATT TGT GC | ATT TCC CTT TCA CCC ACC TCT GTT T | E | M |
| Satt510 | GCG AGT TTC GCC GTT ACC ACC TCA GCT T | CCC TCT TAT TTC ACC CTA AGA CCT ACA A | F | M |
| Satt308 | GCG TTA AGG TTG GCA GGG TGG AAG TG | GCG CAG CTT TAT ACA AAA ATC AAC AA | M | P |
| Satt337 | GCG TAA ATC TGA TAT ATG TTA CCA CTG A | GCG TAA TAC GCA AAA CAT AAT TAG CCT A | K | P |
| Satt373 | TCC GCG AGA TAA ATT CGT AAA AT | GGC CAG ATA CCC AAG TTG TAC TTG T | L | M |
| Sat_195 | GCG ATT ACC AAC ACA GGA ACC TTA TA | CGC GAC TCT GAT AAC AAC AAT GTA T | N | M |
| Satt382 | GCG ACA TTA TTG TTT GGA TAG TAA GAT T | GCG CGA TTC TTT TAA ACA ATT CAA ACA C | A1 | M |
| Satt251 | CCT CCA CCC CCT TCC CAC CCA AAA | GGT GAT ATC GCG CTA AAA TTA | B1 | M |
| Satt578 | CCC ACG TCA TAT CCA CTG CTC CTT A | ACA GCA TCG ATA CCA TGA TCT AT | C1 | M |
| Satt406 | GCG TGA GCA TTT TTG TTT | TGA CGG GTT TAA TAG CAT | A2 | P |
| Satt431 | GCG TGG CAC CCT TGA TAA ATA A | GCG CAC GAA AGT TTT TCT GTA ACA | J | P |
| Satt440 | TGA GAA CGT TTG AAA AGA GAT | GAAGAGATTAAGCATAAAGAATACTT | I | P |
| Satt127 | CGC TTG TGA ACC CTG CTA AA | CCA TCC TCT GAA ACC GTT ATC T | I |  |
| Satt076 | TAA TCG AGA TTT AAT AGA AAA CA | TGG ATG GAC ATT TTC AG | L | M |
| Satt562 | GCG GAT TCA CTA GGA TGT TTA T | GCG GCG GCA GCT TAA ATG GAT TGA | I | P |
| Satt484 | GCG TTT AAT AAA ACT AAT TTA ATT GTA CT | GCG TTC CCT TTC TCT CCT TTC TTT CTT | B1 | M |
| Satt288 | GCG GGG TGA TTT AGT GTT TGA CAC CT | GCG CTT ATA ATT AAG AGC AAA AGA AG | G | P |

**M-Monomorphic; P-polymorphic; NA-No Amplification**
